# Innovative Amber-Based Composite—From Mechanochemical Synthesis and Physicochemical Characterization to Application in Cosmetics

**DOI:** 10.3390/ijms26094238

**Published:** 2025-04-29

**Authors:** Małgorzata Wiśniewska, Victoria Paientko, Iwona Ostolska, Karina Tokarska, Natalia Kurinna, Vita Vedmedenko, Olha Konshyna, Volodymyr Gun’ko, Piotr Nowicki

**Affiliations:** 1 Department of Radiochemistry and Environmental Chemistry, Institute of Chemical Sciences, Faculty of Chemistry, Maria Curie-Sklodowska University in Lublin, M. Curie-Sklodowska Sq. 3, 20-031 Lublin, Poland; 2 Chuiko Institute of Surface Chemistry, National Academy of Sciences of Ukraine, 17 Oleg Mudrak Street, 03164 Kyiv, Ukrainevlad_gunko@ukr.net (V.G.); 3 ZELENAHA, 5, Shevchenka Str., 17021 Otrokhy, Chernihiv Region, Ukraine; 4 SPA-Vita Product, 6, Novodarnytska Str., 02097 Kyiv, Ukraine; 5 T-LAB Professional, Heroiv Dnipra, 04214 Kyiv, Ukraine; 6 Department of Applied Chemistry, Faculty of Chemistry, Adam Mickiewicz University in Poznań, Uniwersytetu Poznańskiego 8, 61-614 Poznań, Poland

**Keywords:** amber-based composite, succinic acid, diatomite carrier, composition optimization, molecular modeling, active substances release, cosmetic application

## Abstract

New ways of ensuring sustainable development in various areas of life are being intensively researched. One of the key priorities is to maximize the use of invaluable natural ingredients in cosmetic products while minimizing the negative impact on the environment. In this study, a three-component natural composite based on amber, diatomite, and Phytokeratin^TM^ (hydrolyzed plant protein) was developed using mechanochemical synthesis. The goal was to maximize the release of biologically active substances, such as succinic acid and Phytokeratin^TM^, in aqueous solution. The physicochemical properties of the materials were characterized using Scanning Electron Microscopy (SEM), thermogravimetric (TG) and differential thermogravimetric (DTG) analysis, Fourier Transform Infrared (FTIR) spectroscopy, and Ultraviolet–Visible (UV-Vis) spectrophotometry. Additionally, Density Functional Theory (DFT) was used to perform quantum chemical calculations and characterize molecular interactions in the composite. The optimized composite demonstrated favorable release characteristics and structural properties, confirming its suitability for cosmetic applications. DFT calculations revealed the potential molecular-level interactions between the organic components, indicating the stability and functional integration of the composite. The resulting innovative composite was successfully incorporated into eco-friendly cosmetic formulations, including a solid shampoo bar and a nail conditioner.

## 1. Introduction

Nowadays, significant attention is paid to composite materials of natural origin [1,2]. This fits very well with the trend of green technologies, which use natural substrates to produce specific products, while eliminating the use of aggressive chemical reagents and minimizing energy and water consumption [3,4]. In this context, mechanochemical synthesis is increasingly being used in various fields of chemistry due to its simplicity, high reaction yields and selectivity, solvent-free nature, shorter synthesis times, room temperature conditions, and easy process scalability [5,6,7]. This approach includes phenomena induced by the action of mechanical energy (grinding, abrasion, kneading, cavitation), leading to changes in the chemical and physicochemical properties of the final material. Mechanochemical synthesis is carried out using ball mills, which are equipped with special reaction vessels (also called jars) in which reagents and grinding balls are placed. There are two types of ball mills, which are standard devices for conducting mechanochemical reactions: mixing mills (portable devices used for small-scale synthesis, providing high-energy ball collisions by using cylindrical vessels oscillating horizontally) and planetary mills (portable or large-scale devices (up to 100 L), in which the movement of the balls inside the vessel is a result of the Coriolis force). In the case of the latter, the different velocities between the balls and the vessel lead to the interaction of friction and impact forces, which generate high dynamic energy. The selection of appropriate parameters (including the type of reagents and additives, the properties of grinding balls (size, mass, type of material) and mixing vessels (volume, lining material), the type of ball mills used, as well as the reaction time and vessel oscillation frequency) is crucial for obtaining the optimal result of the synthesis and is most often determined empirically for the selected type of reaction [8,9].

The growing interest in natural materials and ecological products makes amber even more attractive and desirable on the market. This fossilized tree resin is distinguished by its variety of colors, from light yellow, through orange, to deep brown. Thanks to its uniqueness and rarity, amber is valued all over the world both as a raw material for jewelry and as a functional material. Each piece of amber is different, often containing organic inclusions (fossilized organisms). Its electrostatic properties also make it useful in some industrial processes. Furthermore, it is being investigated for its potential applications in optics and electronics. An important and extensive area of amber application is the production of cosmetics, such as creams, shampoos, soaps, balms, and peelings, which are intended to improve the condition of the skin and hair. Amber powder and amber extract are commonly used for this purpose. These components contain valuable chemical compounds that can exhibit antioxidative, antibacterial, antifungal, and antiphlogistic properties, as well as repellent and insecticidal activity [10]. It is worth noting that the majority of these compounds are included in the INCI database, which confirms their regulatory approval and functional relevance in cosmetic applications. These include, among others, limonene, camphor, camphene, cymene, eucalyptol, α-pinene, β-pinene, borneol, and abietic acid [11,12].

An extremely valuable component of natural amber is succinic acid. It is estimated that the fossilized tree resin contains about 8% of this compound [13]. Succinic acid (also known as 1,4-butanedioic acid, 1,2-ethanedicarboxylic acid, or amber acid) belongs to the group of dicarboxylic acids. It takes part in biochemical reactions leading to the formation of ATP—the cellular energy necessary for the proper functioning of cells and activating their metabolism. This compound shows antioxidant, purifying, and moisturizing properties, and is often used in skin care to regulate sebum and reduce imperfections [10,14]. Succinic acid stimulates and improves cell metabolism, thanks to which it reduces wrinkles and adds radiance to tired skin. It is also a strong antioxidant, which neutralizes free radicals that accelerate skin aging. Moreover, it has antibacterial and mild exfoliating properties; its effect on the skin is comparable to that of alpha-hydroxy acids (AHAs), while remaining non-irritating [15,16]. Cosmetics containing succinic acid are intended especially for mature and dry skin requiring intensive revitalization. They visibly improve skin hydration and lubrication, enhancing its firmness and elasticity.

In light of the above, this study focuses on the mechanochemical synthesis of a three-component amber-based composite, its physicochemical characterization, and practical application in cosmetic products for hair and nail care. In addition to amber powder, hydrolyzed plant protein was used as the active ingredient, while diatomite was used as the carrier. The proposed innovative material with an optimized composition is entirely of natural origin, which can make it safer for body care products. However, it should be emphasized that even ingredients of natural origin can have an allergenic, irritating, or comedogenic effect, depending on individual skin characteristics. Furthermore, its presence in wastewater (at later stages) does not pose a threat to the natural environment. The second active ingredient of the composite—Phytokeratin^TM^—is a plant-based alternative to conventional keratin. It is obtained as a hydrolysate from wheat, corn, and soy. Phytokeratin^TM^ exhibits all the beneficial properties of keratin amino acids, which are essential for maintaining the healthy appearance of hair, skin, and nails [17]. In turn, diatomite (the composite carrier) is a natural amorphous diatomaceous earth derived from fossilized diatom shells of *Melosira preicelanica*—a species inhabiting very clean freshwater environments. It is the most absorbable form of organic silicon, distinguished by a very high silica content (approximately 92%) [18].

## 2. Results and Discussion

### 2.1. Optimization of the Amber-Based Composite Content

**Figure 1:**
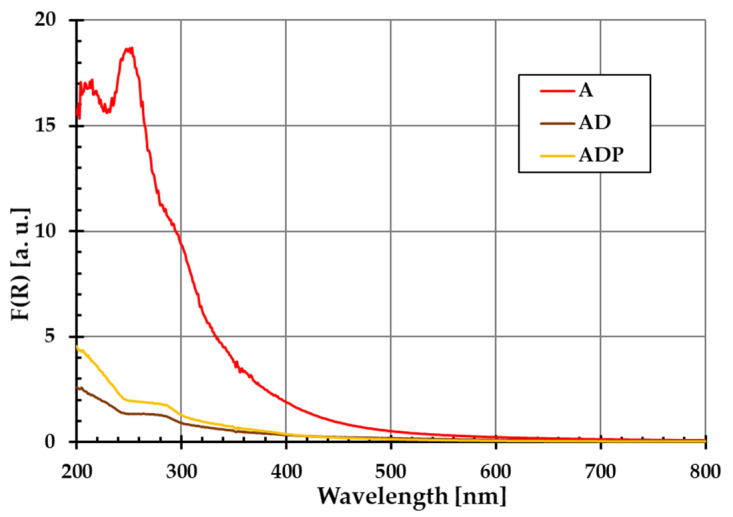
Ultraviolet–visible diffuse reflectance spectra of amber powder (A), amber–diatomite composite (AD), and the three-component amber–diatomite–Phytokeratin^TM^ composite (ADP).

The diffuse reflectance spectra of amber (A), amber–diatomite (AD), and amber–diatomite–Phytokeratin^TM^ (ADP) solid samples are presented in Figure 1. No additional peaks are observed, which confirms that the materials used are characterized by high purity. This is extremely important in the context of their application in the production of safe cosmetic products.

**Figure 2:**
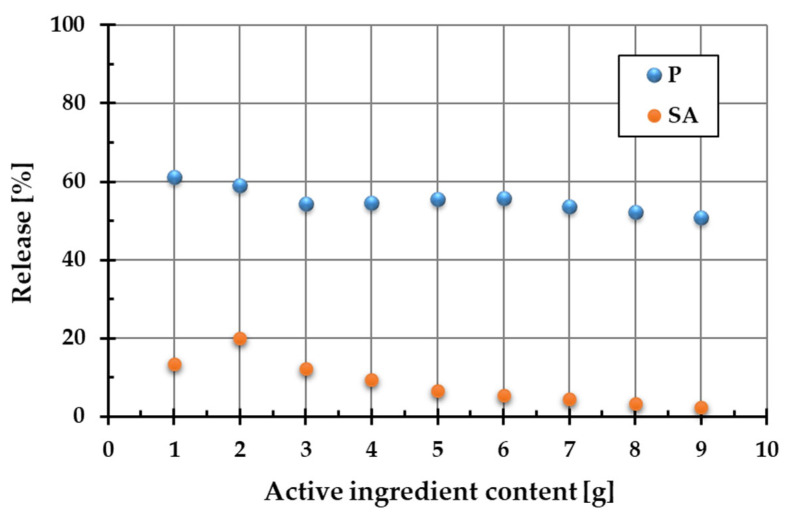
Release of Phytokeratin^TM^ (P) and succinic acid (SA) from binary systems containing diatomite as the carrier.

Figure 2 shows the percentage release of active ingredients (Phytokeratin^TM^ (P) and succinic acid (SA)) into the aqueous phase from the two-component systems containing a diatomite carrier and a variable amount of plant protein or amber.

As demonstrated by the results, the Phytokeratin^TM^ release from the composite materials is considerably higher than that of succinic acid. For plant protein, this parameter changes in the range of 50.7–60.1%, whereas for succinic acid, it varies from 2.4 to 20.1%. This difference can be attributed to the form of the starting ingredients. Phytokeratin^TM^ was present in the composite in pure powdered form, whereas amber is a complex natural mixture containing succinic acid and other constituents. Consequently, the dissolution of plant keratin and its release from the single-component powder was much more effective than the transfer of succinic acid into the aqueous solution from the complex amber matrix. Based on these findings, the optimal composition of ADP material was selected to ensure the highest release of both active ingredients. The optimal proportions turned out as follows: 2 g of amber (20.1% release of SA), 1 g of Phytokeratin^TM^ (60.1% release of protein), and 7 g of diatomite. This material with an optimized composition was used for further research and cosmetics preparation.

It has also been shown that even small amounts of both active substances in the composite ensure their effective release. Increasing the content of hydrolyzed plant protein and amber in the composite structure did not lead to the increased release of these substances into the aqueous solution. This can be attributed to the more intensive hydration of the ingredients, which results in an increasing number of mobile protons (H^+^ ions) in the system. Their presence affects pH changes, which result in conformational changes in these molecules. This in turn affects their release from the composite into the aqueous phase.

### 2.2. Physicochemical Characteristics of Amber-Based Composite

**Figure 3:**
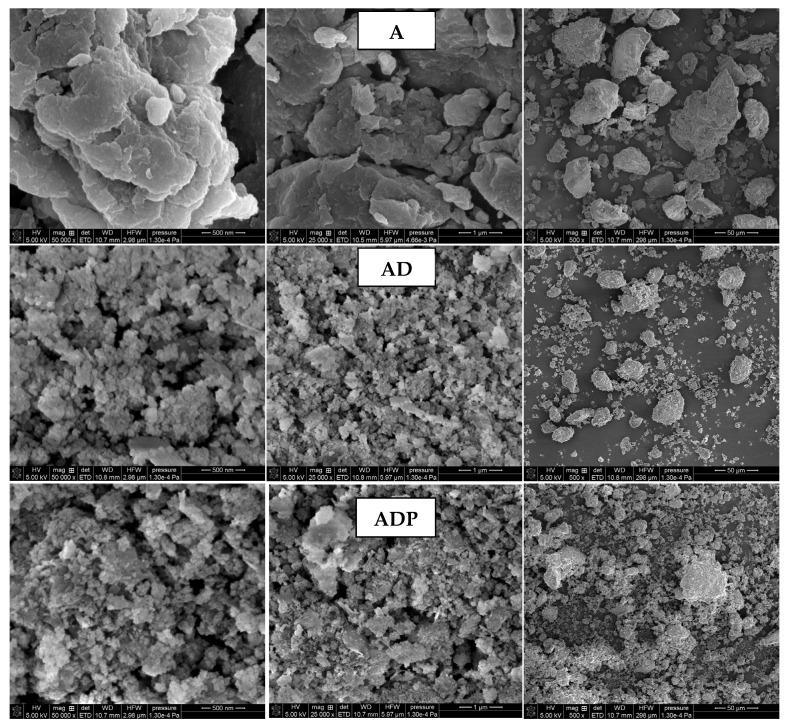
Scanning electron microscopy (SEM) images of amber powder (A), amber–diatomite composite (AD), and the three-component amber–diatomite–Phytokeratin^TM^ composite (ADP) at different magnifications (50,000×, 25,000×, and 500×).

The surface morphology of the samples (A, AD, and ADP) obtained by SEM imaging is presented in Figure 3. A comparative analysis of SEM imaging results indicates that the addition of the diatomite carrier to Niedźwiada amber results in the formation of the AD composite, which is characterized by a reduction in the size of solid particles and the formation of smaller aggregates. This tendency becomes even more visible after the introduction of Phytokeratin^TM^, resulting in the highest solid phase dispersion degree for the three-component ADP composite. The increase in particle dispersion in the final composite is highly desirable as it guarantees its better distribution throughout the entire volume of the cosmetic product [9].

**Figure 4:**
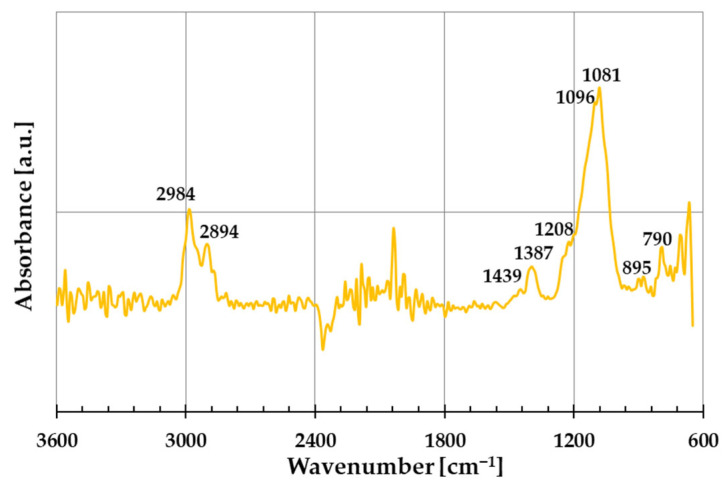
Fourier transform infrared (FTIR) spectrum of the prepared three-component amber–diatomite–Phytokeratin^TM^ composite (ADP).

The FTIR spectrum of the prepared amber–diatomite–Phytokeratin^TM^ composite is shown in Figure 4. These data indicate that the ADP sample exhibits the bands characteristic of the individual components of the composite, most of which unfortunately overlap. Furthermore, the low intensity of some bands may result from the significant predominance of diatomite in the composition of the produced material. The intense maxima observed at 2984 cm^−1^ and 2894 cm^−1^ can be assigned to the asymmetric and symmetric stretching vibrations of C–H bonds in CH_3_ and CH_2_ groups, which may originate from any of the ingredients used to prepare the composite. The weak bands located at 1439 cm^−1^ and 1387 cm^−1^ correspond to C–H bending vibrations in methyl and methylene groups present in amber or Phytokeratin^TM^ [19]. Moreover, the first of these bands can also be assigned to the amino acid residue vibrations present in the structure of the plant protein used. In turn, the very broad and intense band with a maximum at ~1090 cm^−1^ can, with high probability, be attributed to the asymmetric stretching vibrations of Si–O–Si and Si-OH groups, which are characteristic of diatomite [20]. It cannot be ruled out that this band results from the superimposition of signals from individual components, such as the C–O stretching vibrations in esters, alcohols, or carboxylic acids present in amber from Niedźwiada, as well as the amide III band assigned to the combination of the stretching and bending vibrations in Phytokeratin^TM^ [21]. The presence of this intense band may also indicate interactions between the individual components of the ADP composite obtained by the mechanochemical method. In the presented FTIR spectrum, a weak band at 790 cm^−1^ can also be observed, which may be attributed to the symmetric stretching vibrations of Si–OH bonds characteristic of diatomite.

**Figure 5:**
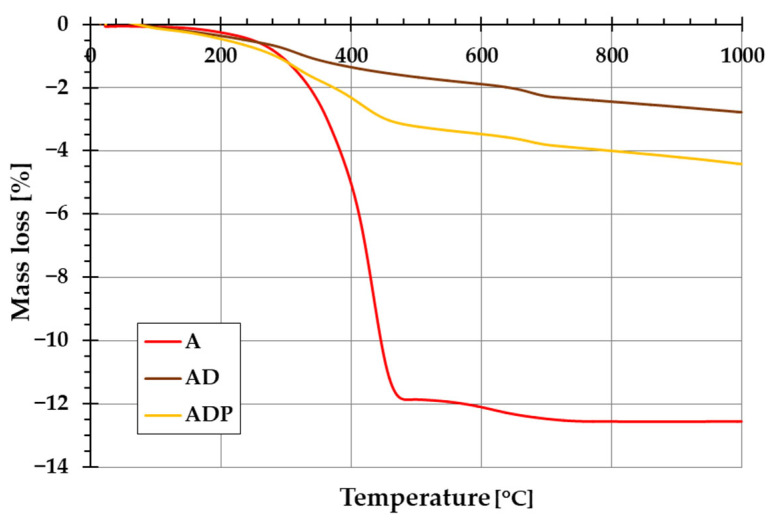
Thermogravimetric (TG) curves of amber powder (A), amber–diatomite composite (AD), and the three-component amber–diatomite–Phytokeratin^TM^ composite (ADP).

**Figure 6:**
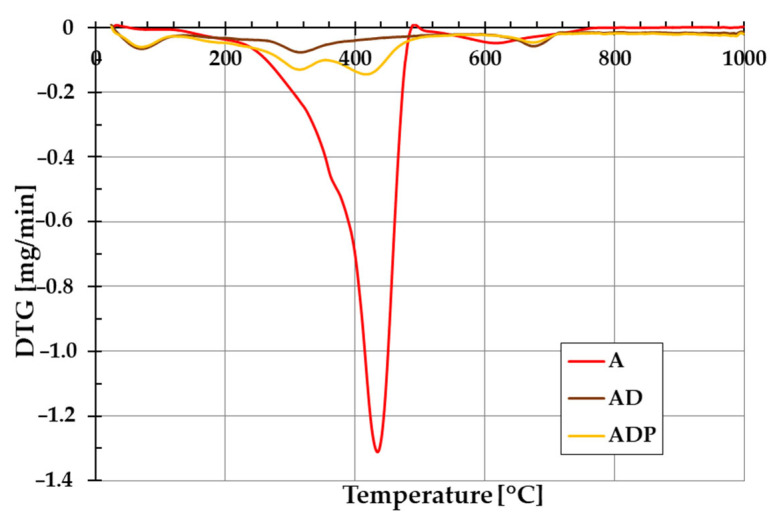
Differential thermogravimetric (DTG) curves of amber powder (A), amber–diatomite composite (AD), and the three-component amber–diatomite–Phytokeratin^TM^ composite (ADP).

The addition of diatomite carrier (and also Phytokeratin^TM^ active ingredient) to amber powder considerably changes the thermal stability of the formed composites. The evidence of this is the course of TG and DTG curves shown in Figure 5 and Figure 6. In the case of amber from Niedźwiada, the total mass loss exceeds 12 wt.%, whereas for AD and ADP composites, it is approximately 2.5 wt.% and 4.5 wt.%, respectively. Thermal decomposition of amber (sample A) is a two-stage process, with the main phase taking place in the temperature range of 120–480 °C, which is associated with the oxidation process of the material (with a significant loss of mass of ~12 wt.%). The intensity of this stage of decomposition significantly depends on the degree of polymerization of the initial amber structure. The higher the degree of polymerization of the resin, the higher the temperature required for its decomposition [19]. In the temperature range of 480–1000 °C, a small mass loss is observed, which is mainly due to the decomposition of the carbon deposits formed after the combustion of the organic matrix from the structures formed in the first stage [22].

In the case of the AD composite, the total mass loss is less pronounced (Figure 5). A small mass loss up to about 120 °C (with a corresponding peak on the DTG curve, Figure 6) is associated with the removal of water physically bound to the silica structures of diatomite. In the temperature range of 200–400 °C, a small mass loss of about 1.75 wt.% occurs, mainly due to the thermal decomposition of amber and the release of the combined water of the clay mineral. At higher temperatures (above 600 °C), similarly to sample A, the observed changes are the result of decomposition of the organic matter of the fossil resin and the superimposed effects of removing the hydroxyl groups of silica and kaolinite degradation [23].

For the ADP composite, similarly to the AD material, the mass loss up to 120 °C results from the removal of physically bound water. In the temperature range of 120–360 °C, a distinct peak on the DTG curve indicates primarily the decomposition of amber. At higher temperatures above 360 °C, Phytokeratin^TM^ is most probably decomposed, and an additional DTG peak becomes visible [24]. Above 440 °C, similarly to the AD sample, the mass loss results from the removal of hydroxyl groups from the silica surface and the combustion of carbon deposits from the amber.

### 2.3. Theoretical Modeling of the Molecular Structure of Composite Components and Their Interactions

Theoretical modeling (with molecular mechanics, MM) of interactions between plant-based keratin and clusters of silica nanoparticles and succinic acid (SA) molecules (Table 1) shows that the hydration energy of polar and ion-generating organics is relatively higher even in the case of incomplete solvation shells with 91–258 water molecules. The solvation of both organics and silica nanoparticles results in a diminution of the interaction energy between them, especially for small acid molecules. In the case of proteins, multi-point interactions with a silica surface can cause stronger adsorption with kinetic inhibition of the desorption, even though the Gibbs free energy of protein adsorption onto a silica surface is not great (about −10 kJ/mol) [25,26,27]. The protein molecules studied are relatively long and typically form triple coils in the native state. Their effective interactions with many nanoparticles, such as fumed silica, clays, etc., can result in strong conformational changes (Table 1, last model) up to the adsorption denaturation [26,27]. Therefore, in the case of practically important systems containing proteins (e.g., collagen, keratin, etc.) and nanooxides, the concentration of nanooxides should be rather low to prevent strong damage to the protein structure, which is a rather negative effect.

**Figure 7:**
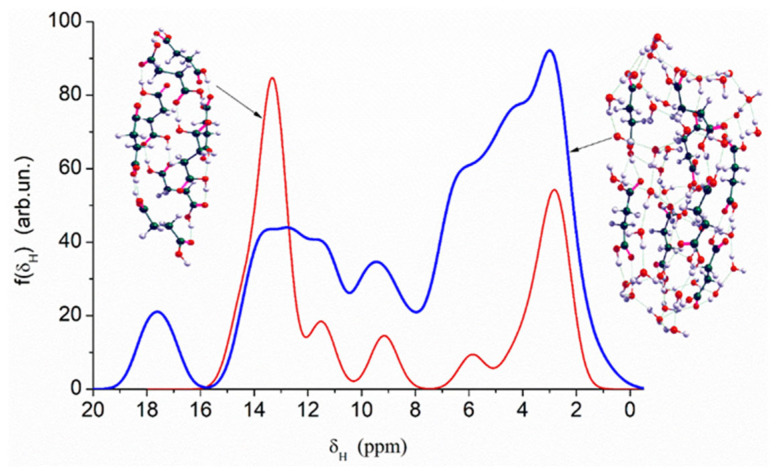
Theoretical ^1^H NMR spectra of the succinic acid cluster (consisting of 8 molecules) in the isolated and hydrated state, calculated using the gauge-independent atomic orbital method (GIAO/ωB97X–D/cc–pVDZ).

**Figure 8:**
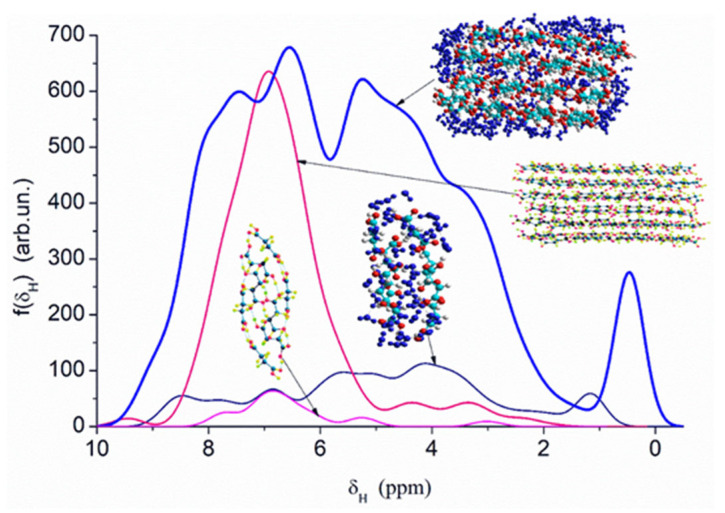
Theoretical ^1^H NMR spectra of succinic acid in the dry and wetted state, calculated using the semiempirical quantum mechanical PM7 method and the correlation function δ_H_ = −27.38435372 + 83.67491184·q_H_ (the spectra include only hydrogen atoms from hydroxyl groups of succinic acid and water molecules, while hydrogen atoms from CH groups are omitted).

**Figure 9:**
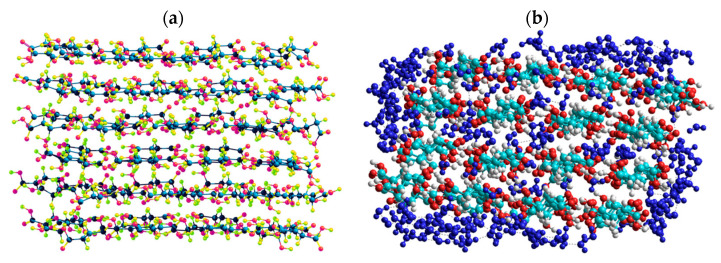
Geometry of a succinic acid cluster consisting of 96 molecules: in the isolated state (**a**) and in the hydrated state with 239 H_2_O molecules (**b**), calculated using the semiempirical quantum mechanical PM7 method.

Quantum chemical calculations with the DFT (Figure 7) and PM7 methods (Figure 8 and Figure 9) show that a decrease in the size of the SA crystallites weakly affects their acidity (Figure 8, curves for dry crystallites) in contrast to the influence of bound water molecules since a downfield shift increases for wetted SA (Figure 7 and Figure 8). Hydration of SA molecules with decomposition of the SA crystallite (Figure 7, right structure, and models of wetted SA in Figure 8 and Figure 9) leads to the appearance of mobile protons, which can form the Eigen (H_3_O^+^) and Zundel (H_5_O_2_^+^) cations (peak at 17.5 ppm). The appearance of the mobile protons (in acidic solutions), characterized by the values of δ_H_ > 10 ppm, could strongly affect the characteristics of water and solutes. An increase in the amounts of water resulting in the dissolution of SA crystallites (Figure 7, Figure 8 and Figure 9, wetted crystallites) can affect the state of water (e.g., pH) and charging of the SA molecules. Since the dissolution of SA crystallites affects the pH value of the solution, the protein molecules could undergo conformational changes strongly sensitive to pH values far from their isoelectric point. Therefore, the amounts of SA, water, and nanosilica tested in the composite systems containing nanooxides and proteins should be rather low to avoid unwanted changes in protein conformation, resulting in changes in the properties of both proteins and composites.

### 2.4. Application of Amber-Based Composite in Cosmetic Products

Using the innovative ADP composite, recipes for two cosmetics for the care of skin appendages (hair, nails) were developed: a solid shampoo bar and a conditioner for the care of nails and the surrounding skin. Aging tests on both products have shown that they have a shelf life of 3 years.

**Figure 10:**
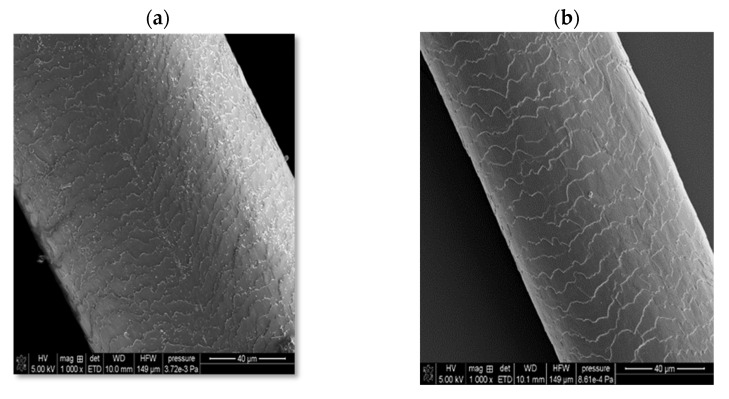

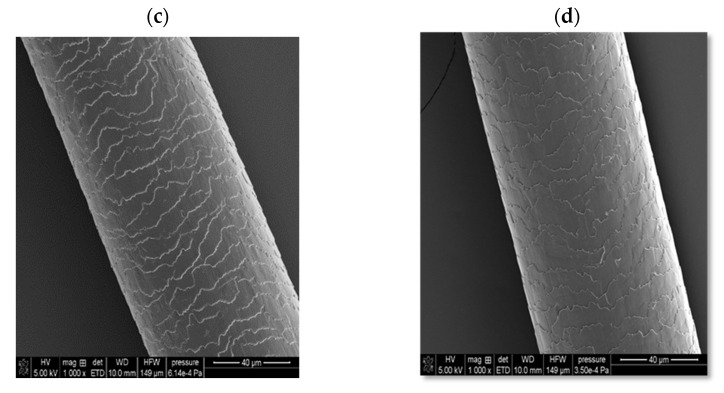
Scanning electron microscopy (SEM) images of hair: before washing (**a**); after washing with a solid shampoo containing a surfactant base without the amber–diatomite–Phytokeratin^TM^ composite (**b**); after washing with a solid shampoo containing an amber–diatomite–Phytokeratin^TM^ mixture (**c**); and after washing with a solid shampoo containing the amber–diatomite–Phytokeratin^TM^ composite obtained via mechanochemical synthesis (**d**).

In the case of solid shampoo, its effectiveness was confirmed by washing model hair with a 5% solution of this cosmetic for 2 weeks. In these experiments, a shampoo that did not contain an amber-based composite was also used, as well as a mixture with a composition analogous to the ADP composite but not subjected to ball mill processing. The SEM images of the examined hair samples are presented in Figure 10.

As can be seen, the initial hair sample (before washing with the solid shampoo, case (a)) is characterized by very open hair scales, whereas in the case of using a cosmetic containing the ADP composite (case (d)), the best hair smoothing effect is achieved. The unique combination of four surfactants, several oils, and an innovative three-component composite containing amber has resulted in the development of a hair care product with many desirable properties, including cleansing, regenerating, nourishing, and smoothing effects. Moreover, it has a convenient cube shape, which greatly facilitates its effective use, convenient storage, and transport.

**Figure 11:**
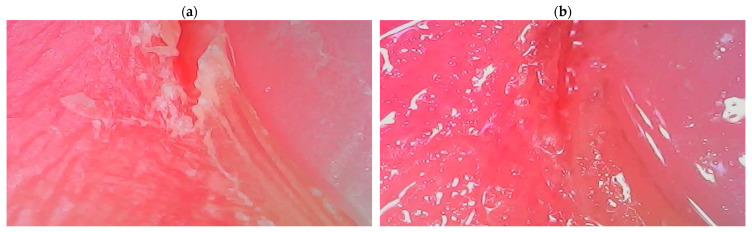
Images of the skin around the nails before (**a**) and after (**b**) application of a nail conditioner containing the amber–diatomite–Phytokeratin^TM^ composite, taken with a dermatoscope.

The second developed product containing the ADP composite is a conditioner for the care of the nail area, the effectiveness of which is confirmed by the dermatoscope images shown in Figure 11.

Its application causes a visible improvement in the skin’s condition, primarily through intense moisturizing. The optimized composition of the nail conditioner (which also contains a unique blend of waxes and oils) ensures multidirectional action on the skin around the nails by oxygenating, regenerating, and nourishing it. The creamy consistency of this preparation enables easy application, resulting in the smoothing and unification of color and the elimination of all imperfections in the skin of the hands.

Calculations performed based on the composition of the amber shampoo bar and nail conditioner using the Rana program [28] showed that these products are characterized by low levels of carcinogenicity, toxicity (including immunotoxicity), and allergy-inducing potential. Therefore, these formulations have the potential for practical application in commercial products. However, some limitations of the studies should be acknowledged. The experiments were conducted exclusively under laboratory conditions, which do not fully reflect the complex and dynamic environment of the human scalp or nails.

Consequently, the findings may not directly translate to real-world applications. Moreover, the short observation period precluded the assessment of long-term effects, such as cumulative damage or sustained improvements. Therefore, before implementing the obtained composite into commercial cosmetic products, additional studies are necessary. These include evaluating its effectiveness and long-term effects, assessing its safety under real-life conditions, conducting consumer tests and sensory evaluations, and ensuring the repeatability of its physicochemical properties. Furthermore, the cost-effectiveness of scaling up production to an industrial level must also be assessed.

## 3. Materials and Methods

### 3.1. Synthesis of Amber-Based Composite

Amber (A), the main component of the composite, comes from amber-bearing formations located in the Lublin region near Niedźwiada (Poland). These deposits were formed approximately 45 million years ago during the Eocene epoch. In the area of today’s Central Europe, there was then a shallow and warm epicontinental sea—the Northern Sea. Its shores were covered with lush forests, producing large amounts of resin, which was later transported by river networks and surface runoff to the coastal zone. As a result of bio- and geochemical processes occurring in seawater, the resin underwent diagenesis and was transformed into amber. Amber-bearing formations in the Lublin region occur at various depths, most often between 15 and 20 m below the ground surface. The size and appearance of Lublin amber lumps vary significantly and occur in three varieties: transparent, translucent, and opaque, with colors ranging from light yellow to brown. The smallest available fraction with a grain size in the range of 0–5 mm was used for the study. It was purchased from STELLARIUM Sp. z o.o., which manages the Open-Air Amber Mine in Niedźwiada–Kolonia. The fine-grained amber was ground into powder using a ball mill (PM 100, Retsch, Verder Polska Sp. z o. o., Katowice, Poland).

Phytokeratin^TM^ (P) was used as the second active ingredient of the composite. This natural protein, in powder form (Aroma-Zone, Cabrières-d’Avignon, France), is obtained by the enzymatic hydrolysis of wheat proteins. It is a plant-based alternative to animal keratin—a fibrous and structural protein. Phytokeratin^TM^ is widely used in the cosmetic industry because its amino acid composition resembles the keratin naturally found in human skin, hair, or nails. The hydrolyzed form of Phytokeratin^TM^ is highly soluble in water and insoluble in oil. The molecular weight of this plant-derived protein ranges from 0.3 to 10 kDa [29].

Diatomite powder (D) was used as a carrier for biologically active substances. The diatomaceous earth (Diato Nat, Mineral Guard Europe, Kąty Węgierskie, Poland) is a soft sedimentary rock composed mainly of fossilized shells of diatoms—single-celled algae. It is rich in health-promoting, easily digestible minerals.

Amber-based composites with different contents of amber, diatomite, and Phytokeratin^TM^ were prepared using the mechanochemical method realized in a ball mill with agate balls and vessel (PM 100, Retsch, Verder Polska Sp. z o. o., Katowice, Poland). The grinding process was conducted for 20 min at a speed of 440 rpm. To optimize the content of active ingredients, two series of two-component composites were initially prepared, each containing 1, 2, 3, 4, 5, 6, 7, 8, and 9 g of amber or plant-based keratin, respectively. The total weight of each sample was 10 g, with the remaining mass supplemented by an appropriate amount of diatomite. Next, 0.05 g of the prepared composites was added to 10 cm^3^ of doubly distilled water, shaken in a water shaker (OLS 200, Grant, Essex, UK) for 5 min, and centrifuged twice (MPW 233e, Med. Instruments, Warsaw, Poland). The concentrations of succinic acid (SA, released from amber) and Phytokeratin^TM^ (P) in the aqueous solutions were determined spectrophotometrically using a Cary 100 Bio UV-VIS spectrophotometer (Varian, Markham, ON, Canada) at a wavelength of 202 nm for SA [30] and 275 nm [31] for Phytokeratin^TM^. Calibration curves showing the dependence of absorbance on the concentrations of SA and P aqueous solutions were prepared in advance. In this way, the percentage of active ingredients released into the aqueous phase from the composite materials was determined. Based on these results, the optimal composition of the three-component composite (ADP) was established, ensuring maximum release of succinic acid and Phytokeratin^TM^.

### 3.2. Physicochemical Characterization of Amber-Based Composite

The purity of all composite components was assessed using a Cary 100 Bio UV-VIS spectrophotometer (Varian, Markham, ON, Canada) equipped with an integrating sphere, which allows for the measurement of the diffuse reflectance of solid samples. Diffuse reflectance occurs when the surface reflects light in many different directions, giving the surface a matte finish.

The surface morphologies of amber, amber–diatomite, and amber–diatomite–Phytokeratin^TM^ samples were examined by the scanning electron microscopy (SEM) with the Quanta 250 FEG instrument provided by FEI (Waltham, MA, USA) equipped with the detector Octane Elect Plus provided by EDAX (Berwyn, IL, USA).

Thermogravimetric analysis of the aforementioned samples was performed on a SETSYS 12 (Setaram, Caluire, France). The samples with a mass of ~10 mg and a particle size below 0.1 mm were heated from room temperature to 1000 °C (at the rate of 10 °C/min), in a helium atmosphere.

The three-component amber–diatomite–Phytokeratin^TM^ composite was also examined by Fourier transform infrared spectroscopy (FTIR) using a Cary 630 FTIR Spectrometer with an ATR sampling module (Agilent Technologies, Santa Clara, CA, USA).

### 3.3. Molecular Modeling of Composite Components Interactions

Quantum chemical calculations of the clusters using the DFT method were performed with a hybrid functional ωB97X-D and the cc-pVDZ basis set, employing the Gaussian 16 C.02 [32], GAMESS 2023 R2 [33], or AMS 2023.1 [34] program suites. The solvation effects were analyzed using the continuum solvation model known as the SMD method, which is based on the quantum mechanical charge density of a solute molecule interacting with a continuum description of the solvent [33,34,35]. The Gibbs free energy of solvation (Δ*G*_s_) was computed as Δ*G*_s_ = *G*_l_ − *G*_g_, where *G*_l_ and *G*_g_ are the Gibbs free energies of a molecule free or bound to a silica cluster in the liquid (subscript l) and gas (subscript g) media, respectively. These calculations included zero-point energy and thermal corrections to the Gibbs free energy in the gas phase and for solved molecules and silica clusters with the geometry optimized using ωB97X-D/cc-pVDZ. NMR spectra were calculated using the gauge-independent atomic orbital (GIAO) method, both with and without the SMD method [32,33,34]. Larger cluster models were calculated using the molecular mechanics (MM) [34,35,36,37] and semiempirical quantum mechanical PM7 method (MOPAC, ver. 22.1.1) [38]. The preparation of initial cluster structures and the visualization of the calculation results were carried out using several software tools [39,40,41,42]. In all the cases, the system geometry was optimized. The structure of plant-based keratin models was based on the PDB data [43]. A silica cluster includes 129 units. The *δ*_H_ values were calculated as the difference in the isotropic values of the magnetic shielding tensors of hydrogen atoms (σ_H,iso_) in TMS (δ_H,TMS_ = 0 ppm), which was used as a reference (e.g., σ_H,iso_ = 31.40 ppm for TMS by GIAO/ωB97X-D/cc-pVDZ) and the given compound [32]. Larger structures were calculated using the PM7 method (MOPAC 2022.1.1) [39]. To calculate the *f*(*δ*_H_) functions using PM7 data, a calibration function with atomic charges *q*_H_ (PM7) and the *δ*_H_ values (GIAO/ωB97X-D/cc-pVDZ) for water clusters calculated by both methods was used *δ*_H_ = −27.38435372 + 83.67491184·q_H_ [25,44].

### 3.4. Application of Amber-Based Composite in Hair and Nail Care Cosmetics

The amber-based composite with an optimal content of active ingredients was used for the preparation of two cosmetic products: a solid shampoo and a nail conditioner. To confirm the safety of the formulas of both cosmetics, calculations were carried out using the specialized proprietary Rana program, developed by Roman Kinash and Victoria Paientko. It is an information system designed to store and systematize the data on prescription compositions, related calculation of the production of certain cosmetics, as well as determining their level of safety in terms of component composition [28]. The exact composition of these formulations is protected by trade secrets [45,46].

The effectiveness of the shampoo was assessed based on washing tests conducted on samples of natural blond hair (Sensationnel, London, UK). This was based on two international cosmetics standards (ISO): (1) ISO 22716:2007—describing good manufacturing practices (GMP) for cosmetic products [47], which includes methods for testing and evaluating the effects of cosmetics on hair, and (2) ISO 16128 (Parts 1 and 2)—defining methods for evaluating natural cosmetic ingredients, including testing shampoos on hair [48,49].

The tests were performed using a 5% aqueous solution of shampoo, prepared using distilled water. This concentration of solid shampoo corresponds to its approximate content in a water bath during hair washing. The hair was cleansed in this solution for 5 min, then rinsed with distilled water and allowed to dry at room temperature. These tests were conducted for a period of two weeks, and individual washings were performed every 2 days. SEM imaging of hair was carried out before and after treatment with the solid shampoo.

The positive effect of the conditioner on nails and the surrounding skin was assessed using a portable dermatoscope (Neviscope, White).

## 4. Conclusions

A three-component composite of entirely natural origin, based on amber, diatomite, and Phytokeratin^TM^ (ADP), was successfully developed using an environmentally friendly mechanochemical synthesis method. Its composition has been optimized to ensure the efficient release of biologically active substances (succinic acid and Phytokeratin^TM^) into the aqueous phase.

The physicochemical analyses confirmed the high purity of the composite, the presence of molecular interactions between its components, and its favorable thermal and morphological properties, including high dispersion and surface modification. These features increase the stability of the material and may improve the bioavailability of active ingredients when applied topically on hair or nails.

The developed innovative amber-based composite has been successfully implemented in the commercial cosmetics formulations for hair and nail care, including solid shampoo bars and nail conditioner, in cooperation with industrial partners such as ZELENAHA, T-LAB Professional, and SPA-Vita Product.

Future studies should focus on assessing the in vivo bioactivity of the ADP composite, its long-term stability within cosmetic matrices, and developing other formulations for various applications. Furthermore, the potential of mechanochemical synthesis as a sustainable and solvent-free method for the development of multifunctional cosmetic ingredients is worth further investigation.

## Figures and Tables

**Table 1:** Interaction energies of hydrated organic compounds (succinic acid, plant-based keratin) with hydrated silica cluster, calculated using the molecular mechanics (MM) method.

Organics	Model	E (kJ/mol)
Succinic acid (8 molecules) interacting with wetted silica nanoparticle	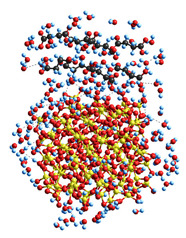	−58.9 ^a^ −5.9 ^b^ −29.5 ^c^
Plant-based keratin (two unfolded molecules) interacting with wetted silica nanoparticle	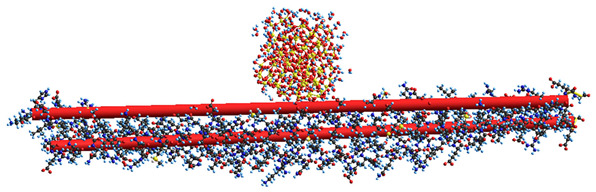	−92.3 ^a^ −6.7 ^b^ −48.0 ^c^
Plant- based keratin (three folded molecules) interacting with wetted silica nanoparticle	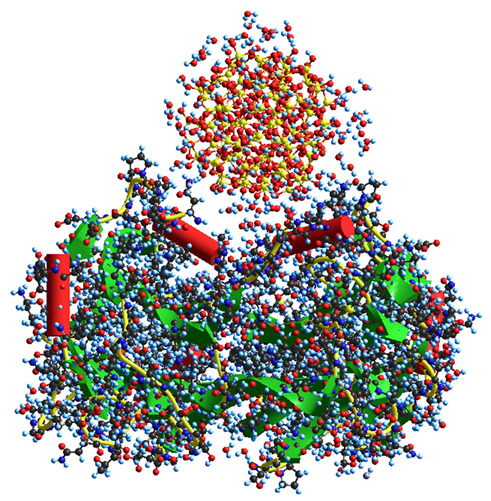	−87.9 ^a^ −6.5 ^b^ −697.5 ^c^

Note: the energy corresponds to interactions between (^a^) organics and silica, (^b^) water and organics/silica per one water molecule, and (^c^) water and organics.

## Data Availability

Data are contained within the article.
